# Emerging significance and therapeutic targets of ferroptosis: a potential avenue for human kidney diseases

**DOI:** 10.1038/s41419-023-06144-w

**Published:** 2023-09-22

**Authors:** Jinghan Li, Sujuan Zheng, Yumei Fan, Ke Tan

**Affiliations:** Ministry of Education Key Laboratory of Molecular and Cellular Biology; Hebei Research Center of the Basic Discipline of Cell Biology, Hebei Province Key Laboratory of Animal Physiology, Biochemistry and Molecular Biology, College of Life Sciences, Hebei Normal University, Shijiazhuang, Hebei China

**Keywords:** Natural products, Cell death, Kidney diseases

## Abstract

Kidney diseases remain one of the leading causes of human death and have placed a heavy burden on the medical system. Regulated cell death contributes to the pathology of a plethora of renal diseases. Recently, with in-depth studies into kidney diseases and cell death, a new iron-dependent cell death modality, known as ferroptosis, has been identified and has attracted considerable attention among researchers in the pathogenesis of kidney diseases and therapeutics to treat them. The majority of studies suggest that ferroptosis plays an important role in the pathologies of multiple kidney diseases, such as acute kidney injury (AKI), chronic kidney disease, and renal cell carcinoma. In this review, we summarize recently identified regulatory molecular mechanisms of ferroptosis, discuss ferroptosis pathways and mechanisms of action in various kidney diseases, and describe the protective effect of ferroptosis inhibitors against kidney diseases, especially AKI. By summarizing the prominent roles of ferroptosis in different kidney diseases and the progress made in studying ferroptosis, we provide new directions and strategies for future research on kidney diseases. In summary, ferroptotic factors are potential targets for therapeutic intervention to alleviate different kidney diseases, and targeting them may lead to new treatments for patients with kidney diseases.

## Facts

- Ferroptosis, which is induced by the accumulation of iron and lipid peroxides, is closely correlated with the occurrence and development of many kidney diseases.
- The application of ferroptosis inhibitors is crucial to the treatment of kidney diseases.
- In-depth study into the molecular mechanisms underlying ferroptosis and regulators of ferroptosis significantly enhances our understanding of the pathologies of kidney diseases.

## Open questions

- How do the identified molecular mechanisms underlying ferroptosis engage in crosstalk?
- What are the effects of the interplay between the ferroptosis pathway and those of other types of cell death on the onset and progression of kidney diseases?
- Are natural small-molecule compounds that target ferroptosis suitable for use in clinical trials?

## Introduction

The cell is the basic unit of life, and its fate and function are influenced by environmental and genetic factors. Because most organisms rely on oxygen as the final electron acceptor in metabolic processes based on reduction/oxidation (redox) reactions, how cells mitigate oxidative stress is a critical factor in cell fate. Among the factors causing oxidative stress in cells, oxidative modification of lipids (especially lipid peroxidation) in the bilayer membrane has been found to be an important regulatory factor that determines cell fate. Excessive lipid peroxidation causes cell death in a unique pattern called ferroptosis. Since the term “ferroptosis” was coined in 2012, the number of studies on this form of cell death has exponentially increased [1, 2].

Ferroptosis is a unique mode of cell death driven by oxidative stress and iron-dependent phospholipid peroxidation. The morphological and biochemical characteristics of ferroptosis are obviously different from those of other forms of regulated cell death (RCD), such as apoptosis and pyroptosis [2, 3]. Morphologically, ferroptosis manifests mainly as loss of cell membrane integrity and blebbing, shrinking mitochondrial cristae and increased mitochondrial bilayer membrane density [2, 4–8]. Loss of membrane integrity during ferroptosis is caused by lipid peroxidation mediated by oxidoreductases, which depend on iron, ROS, and polyunsaturated-fatty-acid-containing phospholipids (PUFA-PLs). Recent studies have shown that the mechanism underlying ferroptosis involves an imbalance of the cell redox system caused by iron-mediated toxic phospholipid hydroperoxide (PLOOH) metabolism disorders, which eventually cause oxidative damage to the cell membrane and proteins; therefore, iron is crucial to ferroptosis [9]. In addition, ferroptosis is orchestrated by a variety of cellular metabolic pathways, including redox homeostasis, iron metabolism, mitochondrial activity, amino acid and lipid and sugar metabolism, and various signaling pathways [7, 10]. Ferroptosis is involved in the pathologies associated with injuries to many organs and degenerative diseases [11, 12]. Interestingly, ferroptosis exerts dual effects by promoting or inhibiting tumorigenesis and tumor growth [13–15]. Therefore, the ferroptosis pathway shows great potential as a target in the treatment of drug-resistant tumors, ischemic organ injury and neurodegenerative diseases, and inducing or inhibiting ferroptosis has become a new strategy to treat human diseases.

## Molecular mechanism underlying ferroptosis

### Abnormal iron metabolism

**Fig. 1:**
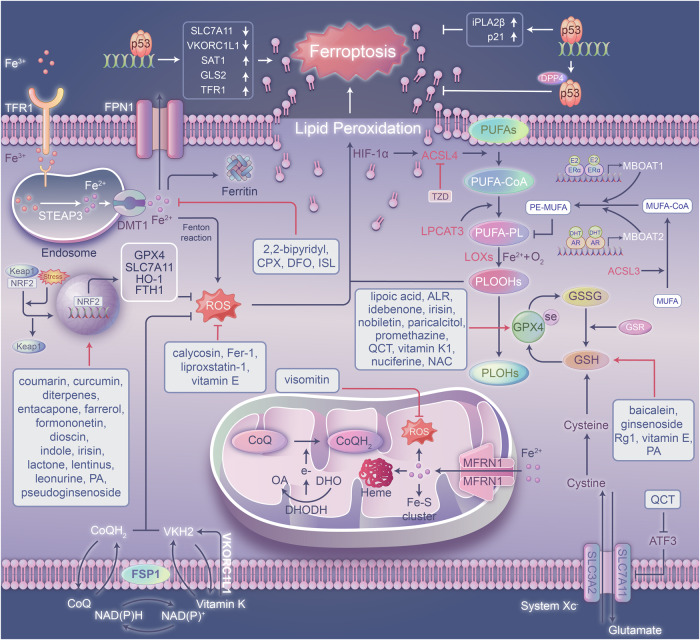
Potential molecular mechanisms and essential coordinators of ferroptosis. The major mechanisms (iron metabolism, ferritinophagy, GSH/system Xc-/GPX4 pathway, FSP1/CoQ/NAD(P)H pathway, and ER/MBOAT1 and AR/MBOAT2 pathways) and important mediators (SLC7A11, GPX4, FSP1, DHODH and VKORC1L1) of ferroptosis are shown. Moreover, other mechanisms and regulators, such as p53, NRF2, ATF3, and HIF1α, are also involved in lipid peroxidation and ferroptosis.

Iron is extensively involved in many metabolic pathways of the human body and in cells with many physiological functions. The steady state of iron metabolism requires that the expression of a series of proteins be maintained accurately and continuously [16, 17]. Iron ions in the form of Fe^2+^ or Fe^3+^ reside in cells *i*n vivo, and iron homeostasis maintains the relatively stable content of these ions through transferrin receptor 1 (TFR1)-regulated iron transport, ferroportin 1 (FPN1)-mediated iron output storage, and ferritin-regulated iron storage (Fig. 1) [18, 19]. When iron homeostasis is imbalanced, ferroptosis is induced. Most intracellular iron exists in the labile iron pool (LIP) [20]. Studies based on changes to the LIP level have revealed that iron overload or ferritinophagy induction increases cell sensitivity to ferroptosis [21–24].

Under physiological conditions, iron binds to proteins such as transferrin, ferritin, and neutrophil gelatinase-associated lipocalin (NGAL) and takes the Fe^3+^ form (Fig. 1). However, under pathological conditions, iron is readily involved a single electron transfer reaction and is transformed into ferrous iron, which shows high reactivity and toxicity. Unstable iron (mainly in the form of ferrous iron) can react with oxygen and produce ROS, such as hydroxyl radicals and hydrogen peroxide, which are related to lipid peroxidation and tissue damage induced through the Fenton reaction. Fe^3+^ is transferred into cells through the membrane protein TFR1 [25, 26]. Ferritin is an iron storage protein complex consisting of ferritin light chain (FTL) and ferritin heavy chain 1 (FTH1) (Fig. 1). Between these two chains, FTH1 exhibits oxidase activity and can convert Fe^2+^ into Fe^3+^. Ferritin integrates iron, which reduces the level of free iron, and thus maintaining the cellular iron concentration [27]. Overexpression of HSP beta-1 (HSPB1) can reduce the intracellular iron concentration by inhibiting TFR1 expression, thus inhibiting ferroptosis (Fig. 1) [28]. Nuclear receptor activator 4 (NCOA4) can bind to ferritin and deliver it to lysosomes for degradation, thereby increasing the iron concentration in cells and ultimately promoting ferroptosis (Fig. 1) [21, 22]. Because cellular iron output is mediated by FPN1 and polycupric ferrous oxide enzymes (such as ceruloplasmin), a decrease in the expression of FPN1 or ceruloplasmin can increase the sensitivity of cells to ferroptosis [29–32]. Moreover, the pentaspan membrane glycoprotein prominin-2 promotes ferroptosis resistance by facilitating the formation of ferritin-containing multivesicular bodies (MVBs) that transport iron out of a cell [33].

### Autophagy and ferritinophagy

Autophagy, which is a survival mechanism of cells under stress conditions, is an intracellular catabolic process through which cell components are transported to lysosomes for degradation. Autophagy has been highly conserved throughout evolution. The core mechanism underlying autophagy involves more than 30 autophagy-related genes (ATGs). Recent studies have shown that ferroptosis is an autophagy-related process [21, 34, 35]. In mouse embryonic fibroblasts (MEFs), autophagy promotes ferroptosis [21]. In ATG5- or ATG7-knockout MEFs, intracellular Fe^2+^ levels and lipid peroxidation levels were significantly reduced, indicating that ATG5- and ATG7-mediated autophagy contributed to ferroptosis [21]. In addition, knockout of other autophagy-related genes, such as ULK1/2, ATG3, and ATG13, also significantly inhibited erastin-induced ferroptosis. These results suggest that autophagy and ferroptosis are activated within the same timeframe; however, the molecular crosstalk between ferroptosis and autophagy pathways is not fully understood, and the molecular mechanism by which autophagy affects ferroptosis needs further exploration [36].

Ferritinophagy, a type of autophagy regulated by NCOA4, plays a vital role in modulating ferroptosis [21]. A study identified the proteins involved in autophagosomes via quantitative proteomics and found that NCOA4 was highly expressed on autophagosomes [22]. Furthermore, as a specific cargo receptor involved in autophagy, NCOA4 bound ferritin and delivered it to lysosomes for degradation, resulting in the release of free iron and an increase in the level of the LIP in cells (Fig. 1) [22, 37–40]. Under cystine deprivation conditions, ferritinophagy is activated to promote NCOA4-regulated ferritin degradation. When ferritin is degraded, ROS accumulates and ferroptosis is subsequently triggered by the increase in the unstable iron content in cells [41]. Therefore, the ferritinophagy pathway is one of the targets for increasing the sensitivity of tumor cells to ferroptosis [36].

### Lipid peroxidation

Lipid peroxidation is a typical feature of ferroptosis [42]. Any free radical that can extract H atoms from oxidizable substrates (such as PUFAs) can initiate lipid peroxidation; therefore, the abundance and location of oxidizable substrates in cells determine the degree of lipid peroxidation and ferroptosis. PUFAs are components of the cell membrane lipid bilayer and are important targets of lipid peroxidation during ferroptosis. PUFA-PLs have become the main targets of lipid peroxidation in ferroptosis due to the instability of their carbon‒carbon double bonds (Fig. 1) [2]. Among PUFAs, arachidonic acid (AA), adrenergic acid (AdA) and phosphatidylethanolamine (PE) are the main substrates that undergo oxidation [43].

The initiation of lipid peroxidation usually starts from the binding of the acyl chain of a PUFA to a phospholipid (PUFA-PL) in the lipid bilayer; then, the diallyl hydrogen atom is transferred between the carbons of a PUFA-PL carbon‒carbon double bonds to form a carbon-centered radical (PL▪), which reacts with molecular oxygen to produce peroxide radicals (PLOO▪). These peroxide radicals then promote the dehydrogenation of another PUFA to form phospholipid hydroperoxide (PLOOH). When the peroxidation of a PLOOH molecule is not catalyzed by glutathione peroxidase 4 (GPX4) to form the corresponding alcohol (a PLOH), the PLOOH and lipid radicals, especially PLOO▪ and alkoxy phospholipid radicals (PLO▪), continue to drive the dehydrogenation of PUFA-PLs and to react with oxygen through the Fenton reaction to generate a large number of PLOOH molecules [5, 15, 44, 45]. Eventually, a large number of secondary products is produced, including products of lipid peroxide decomposition, such as 4-hydroxynonenoic acid and malondialdehyde (MDA), and various oxidized and modified proteins (Fig. 1). These reactions lead to the destruction of membrane integrity and ultimately accelerate the rupture of organelle and cell membranes [2, 43]. At present, PLOOH is considered the driving force of ferroptosis.

Recently, two membrane remodeling enzymes, acyl-CoA synthetase long-chain family member 4 (ACSL4) and lysophosphatidylcholine acyltransferase 3 (LPCAT3), were identified as important drivers of ferroptosis by genome-wide screening and screening with CRISPR/Cas9 technology [46–49]. PUFAs were activated by ACSL4 to generate PUFA-derived acyl-CoAs, and phospholipid peroxides were produced by LPCAT3 (Fig. 1). Lipid peroxidation is also mediated by the activity of lipoxygenases (LOXs) and increased LOXs-regulated lipid hydroperoxide generation enhances susceptibility to ferroptosis. LOXs and/or cytochrome P450 oxidoreductases (PORs) have been shown to initiate lipid peroxidation through lipid deoxygenation [5, 50, 51]. However, the LOX participation in ferroptosis needs to be verified with genetic evidence.

The overactivation of ACSL4, LPCAT3, and LOXs produces a large amount of phospholipid peroxides, which trigger ferroptosis. Phosphatidylethanolamine-binding protein 1 (PEBP1) and arachidonate 15-lipoxygenase (ALOX15) can interfere with phospholipid alcohol synthesis and induce ferroptosis [5, 52]. ALOXs are nonheme ferric enzymes that catalyze the oxidation of PUFAs to generate a series of secondary metabolites that in turn promote ferroptosis [27]. However, once iron metabolism is altered, the iron concentration is increased, which is conducive to ALOX activation. ALOX15 can bind to PEBP1 and mediate RSL3-induced ferroptosis in bronchial epithelial cells, renal epithelial cells, and neurons [52]. ALOX5 is also involved in ferroptosis and can be inhibited by its binding protein microsomal glutathione S-transferase 1 (MGST1) [53]. GPX4 plays a vital role not only in the system Xc- pathway but also in the lipid peroxidation pathway [54, 55]. GPX4 can oxidize glutathione (GSH) to oxidized glutathione (GSSG) and reduce lipid peroxides to the corresponding alcohols (Fig. 1) [45, 56]. In addition, heat shock proteins (HSPs) inhibit lipid peroxidation and interfere with ferroptosis. For example, the phosphorylation of HSPB1 affects iron absorption and subsequent lipid ROS production; thus, overexpression of HSPB1 inhibits ferroptosis [28].

In addition to lipid peroxidation, ferroptosis inducers can cause DNA damage, which further aggravates ferroptosis. Erastin and sorafenib induce the expression of γ-H2AX, which damages DNA [27, 57]. During ferroptosis, the increase in the DNA oxidation rate not only increases genomic instability but also leads to autophagy. ATM and ATR are serine/threonine kinases that repair DNA damage and alleviate ferroptosis by phosphorylating corresponding downstream proteins [58].

## Mechanism of defense against ferroptosis

With the in-depth study of ferroptosis, at least three types of ferroptosis defense systems have been identified based on different subcellular localizations in cells, including GPX4 in the cytoplasm and mitochondria, DHODH in mitochondria and FSP1 on the cell membrane; these systems drive tripartite activity to defend against ferroptosis [10]. Moreover, NRF2, p53, and other factors play crucial roles in ferroptotic cell death [59, 60]. Recently, vitamin K was unexpectedly identified as a factor in the defense against ferroptosis [61, 62].

### GSH/system Xc-/GPX4 pathway

GSH is a tripeptide composed of three amino acids (cysteine, glutamate, and glycine) and is one of the most abundant antioxidants in cells [63]. Cystine is an amino acid essential for glutathione synthesis. Intracellular glutamate and extracellular cystine are exchanged in equal proportions [5, 64]. Cystine enters cells through system Xc- and is reduced to cysteine via the thioredoxin reductase 1 (TXNRD1)-dependent pathway and then contributes to GSH production (Fig. 1). Inhibition of cystine input inhibits the synthesis of GSH in cells to the large extent and induces ferroptosis. The system Xc- transporter is a target upstream of the ferroptosis pathway [10, 46, 65–67]. Solute carrier family 7 member 11 (SLC7A11) is the main active subunit of system Xc-, and it regulates the dynamic GSH level to maintain its equilibrium and thus regulate ferroptosis [7, 68].

GPX4 was first purified by Ursini and colleagues through biochemical technology [69]. GPX4 is one of the strongest antioxidant enzymes in the human body and belongs to the glutathione peroxidase family. As a selenoprotein, GPX4 is the main catalytic enzyme that mediates PLOOH reduction and detoxification in mammalian cells. GSH is a powerful reducing agent that functions as a cofactor of GPX4 and promotes the reduction of PLOOHs into their corresponding alcohols (PLOHs) in cells, thereby protecting the cell membrane (Fig. 1) [7, 43, 70]. GSH-disulfide reductase (GSR) reproduces GSH by oxidizing glutathione (GSSG) with electrons provided by NADPH/H^+^ [71].

When the activity and/or expression system Xc- is inhibited, intracellular GSH biosynthesis is hampered, and lipid ROS accumulate, which eventually induces ferroptosis. In the classical ferroptosis regulatory pathway, GSH is depleted by erastin or other system Xc- inhibitors, which reduces cysteine synthesis and leads to GSH synthesis disorder [2, 72, 73]. Obstruction of GSH synthesis affects the lipid membrane repair ability of GPX4, increases the rate of toxic lipid free radical and ROS accumulation, and promotes lipid peroxidation, resulting in ferroptosis [1, 2]. In addition, RSL3, another ferroptosis inducer, can covalently bind to selenocysteine at the active site of GPX4, thus directly inhibiting the phospholipid peroxidase activity of GPX4 [54]. In addition, other compounds, such as ML162, withaferin A (WA), the FDA-approved anticancer drug altretamine and sorafenib, can induce ferroptosis by inactivating GPX4 [54, 74–78].

### Mitochondrial pathway

GPX4 has been considered an indispensable protein that regulates ferroptosis. Recently, the system of defense against ferroptosis in mitochondria has been revealed, and it has been suggested to be a novel mechanism that regulates ferroptosis independent of the GPX4 pathway [79]. Mitochondria are organelles with double-membrane structures in eukaryotic cells and are the main sites of aerobic respiration [80]. Moreover, mitochondria, as “power houses” of cells, are critical for producing ATP. During oxidative phosphorylation, the electron transport chain in the inner membrane produces a large number of ROS [81]. When the mitochondrial antioxidant system is damaged and ROS cannot be eliminated from cells, lipid peroxidation is mediated through the Fenton reaction [7, 82]. Recently, an interesting study identified dihydroorotate dehydrogenase (DHODH) as a novel ferroptosis suppressor that functions as independent of the classical GPX4 signaling pathway and revealed the mitochondrial lipid peroxidation-dependent ferroptosis pathway [79]. DHODH is a flavin-dependent enzyme located in the inner mitochondrial membrane. Its main function is to catalyze the fourth step of the pyrimidine biosynthesis pathway, namely, the oxidation of dihydroorotate (DHO) to yield orotate (OA) and transfer electrons to ubiquinone in the inner mitochondrial membrane at the same time, so OA can be reduced to dihydroubiquinone (Fig. 1) [83, 84]. DHO and OA supplemented with DHODH can attenuate and enhance ferroptosis, respectively [79]. In addition, the inactivation of DHODH can aggravate ferroptosis, which indicates that DHODH shows a profound ability to counteract ferroptosis [79]. Increasing DHODH expression or inhibiting its degradation rate has become an effective way to inhibit ferroptosis. A recent study showed that adenylate kinase 2 (AK2) phosphorylated lysyl-oxidase 3 (LOX3) at S704 and thus stabilized its structure and increased its lysyl-oxidase activity [85]. As a result, ubiquitination and degradation of DHODH were inhibited, and the mitochondrial ferroptosis pathway was subsequently blocked [85]. Thus, the combination of DHOHD inhibitors with chemotherapy drugs is expected to become a new treatment strategy for drug-resistant tumors.

After discovering DHODH, the same group identified another inner mitochondrial membrane-bound enzyme, glycerol-3-phosphate (G3P) dehydrogenase 2 (GPD2); this enzyme is a novel inhibitor of ferroptosis that blocks mitochondrial lipid peroxidation and promotes the reduction of CoQ to CoQH_2_ in mitochondria [86]. In summary, DHODH and GPD2 prevent ferroptosis in the mitochondria by regulating the production of dihydroubiquinone in the inner mitochondrial membrane; this is a novel ferroptosis regulation pathway that parallels the GSH/system-Xc/GPX4 axis.

### The FSP1/CoQ/NAD(P)H pathway

Ferroptosis suppressor 1 (FSP1, formerly known as AIFM2) has been identified as another ferroptosis inhibitor independent of the GPX4 pathway that mainly functions on the cell membrane [87, 88]. Overexpression of FSP1 significantly reduces ferroptosis caused by GPX4 inhibition. Myristoylation at the N-terminus of the FSP1 protein, a lipid modification, facilitates the localization of FSP1 to the plasma membrane; this is an important step for its anti-ferroptosis activity. Mechanistically, myristoylation promotes the recruitment of FSP1 to the plasma membrane, where it functions as an oxidoreductase, mediates the reduction of NADH-dependent CoQ, and produces a lipophilic free radical-trapping antioxidant (RTA) that inhibits the lipid peroxidation chain reaction [6, 11, 88]. Specifically, the inhibition of ferroptosis by FSP1 is mediated by ubiquinone, also known as coenzyme Q_10_ (CoQ_10_). FSP1 can inhibit ferroptosis by reducing ubiquinone or CoQ to ubiquinone or CoQH_2_ on the cell membrane, with the latter functioning as an antioxidant that trap radicals and thus prevents lipid peroxidation, thereby preventing ferroptosis (Fig. 1) [87–89]. Hence, inhibition of FSP1 activity has emerged as a promising strategy in cancer treatment because it triggers ferroptosis. iFSP1 has been identified as a specific inhibitor of FSP1 [87]. Following iFSP1 treatment, the sensitivity of tumor cells to ferroptosis was obviously enhanced, suggesting a new strategy for tumor treatment. In addition to iFSP1, a compound in the class of 3-phenylquinazolinones (called icFSP1) was identified as an FSP1-specific inhibitor that induces the subcellular translocation, condensation, and phase separation of FSP1 [90]. Intrinsically disordered regions (IDRs) and low-complexity regions (LCRs) in FSP1 and myristoylation of FSP1 are essential for its phase separation. icFSP1 also represses tumor growth and causes FSP1 undergo condensation in tumors [90]. These results suggest that targeting FSP1 or inhibiting its phase separation initiates ferroptotic cell death is a potential anticancer paradigm.

### ER/MBOAT1 and AR/MBOAT2 signaling pathways

Recently, membrane-bound O-acyltransferase domain-containing 2 (MBOAT2) was identified as a ferroptosis-suppressing gene through whole-genome CRISPR activation screening (Fig. 1) [91]. MBOAT1 also exhibited an anti-ferroptotic effect in a similar manner independent of GPX4 and FSP1. Mechanistically, MBOAT1/2, which are lyso-PL acyltransferases (LPLATs), change monounsaturated fatty acids (MUFAs) into lyso-phosphatidylethanolamines (lyso-PEs), increasing the levels of PE-MUFAs and decreasing the levels of PE-PUFAs to suppress phospholipid peroxidation and inhibit ferroptosis [91]. In addition, MBOAT1 and MBOAT2 are transcriptionally regulated by the estrogen receptor (ER) and androgen receptor (AR), respectively (Fig. 1). In addition, treatment with enzalutamide (ENZ) and ARV-110, two anti-AR drugs, enhance the sensitivity of AR+ prostate cancer cells to ferroptosis by decreasing the expression of MBOAT2 [91]. In ER+ breast cancer cells, treatment with the ER degrader fulvestrant markedly elevated cell sensitivity to ferroptosis by downregulating MBOAT1 expression [91]. This study illustrated for the first time that sex hormone signaling contributes to the ferroptosis pathway by regulating the expression of different genes, providing a new molecular biology explanation for gender differences in the incidence rate of kidney disease. Whether there is signaling crosstalk established between ER and AR or whether they inhibit ferroptosis through other unknown target genes are interesting topics for future studies. In addition, the effects of AR and ER activation on kidney disease need to be further clarified.

### The p62-Keap1-NRF2 pathway

NRF2 is a stress-inducible transcription factor. Under physiological conditions, the cytoplasmic protein Kelch-like ECH-associated protein 1 (Keap1), an adapter protein of Cullin-3-based ubiquitin ligase, binds to NRF2 to suppress NRF2 activation by mediating its ubiquitination and degradation (Fig. 1) [92, 93]. After exposure to electrophilic or oxidative stress, the sensor residues cysteine of Keap1, especially C151, C226, C273, and C288, undergo oxidation and modification, causing conformational changes that inhibit Keap1-mediated NRF2 degradation [94]. As a result, NRF2 is freed from Keap1, with which it forms a complex, and is translocated into the nucleus where it binds to antioxidant responsive elements (AREs) in the promoters of target genes (Fig. 1) [92–94]. Numerous studies have identified many target genes of NRF2 and revealed functions of pleiotropic NRF2 in addition to its redox-regulating function. Moreover, as an adapter protein involved in selective autophagy and a target of NRF2, p62/SQSTM1 competitively binds to Keap1, subsequently promoting NRF2 activation. Phosphorylation of p62 significantly increases the binding affinity of p62 for Keap1 [95, 96]. Therefore, the p62-Keap1-NRF2 axis forms a feedback loop that positively regulates the cytoprotective effects of NRF2.

NRF2 is considered the main regulator of the antioxidant response because many of its downstream target genes are involved in preventing or correcting redox imbalance in cells [97]. Proper NRF2 function is essential for cell survival, especially those under oxidative stress or iron homeostasis imbalance conditions. Therefore, NRF2 plays an important role in maintaining the cell redox balance and preventing ferroptosis. The pathways of NRF2 activity that inhibit ferroptosis can be classified into the following three categories: iron/heme metabolism, GSH synthesis/metabolism, and regulation of intermediate metabolite production pathways (Fig. 1) [98, 99]. The transcription of a set of genes related to the regulation of heme synthesis and transformation, such as heme oxygenase 1 (HO-1), ferrochelatase (FECH) and SLC48A1, is upregulated by NRF2 [100–103]. These findings imply that NRF2 is of great importance for maintaining iron/heme homeostasis [99, 104]. In addition to iron and heme, numerous genes associated with GSH synthesis and metabolism are controlled by NRF2. GPX4, glutathione synthesis gene γ-glutamylcysteine synthase (GCS), GCLC, GCLM, and SLC7A11 are known targets of NRF2 [98, 105–107]. Thus, activation of NRF2 is expected to protect cells from ferroptosis. In addition, NRF2 can also regulate the transcriptional expression of metabolites involved in intermediate metabolism, some of which play essential roles in the regeneration of NADPH, a key electron donor needed to reduce oxidative substrates [108]. However, some findings have demonstrated that NRF2 positively regulates other pathways to induce ferroptosis. For example, HO-1 shows dual abilities and can promote or inhibit ferroptosis [109]. Overactivation of NRF2 promotes HO-1-mediated and iron-catalyzed generation of ROS and induces ferroptosis [109–111]. Thus, it is important to understand in detail the transcriptional mechanism underlying NRF2 regulation of the expression of different target genes under different conditions and tissues to make full use of the ability of NRF2 to defend against ferroptosis and be leveraged to treat human diseases.

### The vitamin K pathway

Vitamin E is considered as the most potent lipophilic antioxidant that traps free radicals. At the beginning of the 21st century, vitamin K was shown to inhibit the downregulation of glutathione and lipoxygenase-dependent oxidative cell death, suggesting a potential correlation between vitamin K and ferroptosis [112, 113]. A recent interesting study discovered that three major forms of vitamin K, menaquinone-4 (MK4), phylloquinone, and menadione, effectively alleviated GPX4 depletion-induced ferroptosis [61]. In addition, MK4 treatment significantly inhibited lipid peroxidation in the mouse liver, prolonged the life of mice with GPX4 deleted and raised under vitamin E-deficient conditions, and protected against tissue injury in a mouse kidney ischemia‒reperfusion model [114]. The activity of FSP1 is important for vitamin K to inhibit ferroptosis in vivo and in vitro [61, 62]. In the nonclassical vitamin K cycle, FSP1 reduces vitamin K to hydroquinone vitamin K (VKH2) (Fig. 1). VKH2 can be used as a lipophilic antioxidant to inhibit ferroptosis by trapping oxygen free radicals. Moreover, FSP1 plays an important role in the classical vitamin K cycle, and it mitigates the toxicity and side effects of warfarin by mediating the reduction of vitamin K [115]. Furthermore, recent CRISPR‒Cas9 knockout screening led to the identification of vitamin K epoxide reductase complex subunit 1-like 1 (VKORC1L1), which is as an important contributor to the defense system against ferroptosis (Fig. 1) [116]. Mechanistically, VKORC1L1 exhibits anti-ferroptosis activity by promoting the generation of the reduced form of vitamin K (vitamin K hydroquinone), thus counteracting phospholipid peroxidation (Fig. 1) [116]. Moreover, the FDA-approved anticoagulant drug warfarin, a small-molecule inhibitor of VKORC1L1, suppresses tumor growth by inducing ferroptotic cell death in vivo, indicating that warfarin may be a potential anticancer drug, especially for cancer patients with high VKORC1L1 expression.

Notably, some studies have shown that vitamin K1 (chlorophylquinone) can compensate for the damage to the anti-ferroptosis defense mechanism when GPX4 was inhibited or when used with the DHODH inhibitor BQR, and its anti-ferroptosis efficacy was found to be equivalent to that of ferrostatin-1 (Fer-1) [61]. In addition, vitamin K1 inhibited ferroptosis of renal tubular cells by reducing the expression of ACSL4 [114]. Thus, the fully reduced form of the vitamin K-a group of naphthoquinones, including methylnaphthoquinone and chlorophylloquinone, robustly protected cells and tissues from ferroptosis. Based on these studies, new treatment strategies can be developed to treat ferroptosis-related diseases.

### p53-regulated ferroptotic pathways

The tumor suppressor protein p53 (TP53), a powerful multifunctional gene, is activated by many types of stresses, such as DNA damage, nutrition deprivation, hypoxia, or oncogene activation [117]. Functioning as a transcription factor, p53 orchestrates various cellular processes, including the cell cycle, cell death and senescence, and exhibits an antitumor function [117, 118]. Unfortunately, p53 is mutated or depleted in approximately 50% of tumors. The relationship between p53 and ferroptosis was reported in 2015 [119]. Subsequently, an increasing number of studies have revealed the complex and contradictory functions of p53 in the regulatory network of ferroptosis (Fig. 1) [60, 120, 121].

#### Ferroptosis-promoting roles of p53

p53 was initially described to be primarily a ferroptosis-promoting factor [119]. p53 inhibits the transcription of SLC7A11 by directly binding to its promoter in fibroblasts and certain cancer cells, which then affects the GPX4-regulated classical pathway and ultimately leads to ferroptosis (Fig. 1) [119]. Mechanistically, p53 decreases the monoubiquitination rate of histone H2B on lysine 120 (H2Bub1) in the SLC7A11 gene regulatory region by facilitating the nuclear translocation of the deubiquitinase USP7, leading to the inactivation of SLC7A11 expression in erastin-treated cells [122]. Thus, the p53-USP7-H2Bub1 axis mediates ferroptosis through an epigenetic mechanism. Interestingly, p53^3KR^, an acetylation-defective mutant form of p53 (K117R, K161R, and K162R), effectively suppressed the expression of SLC7A11 but not other target genes of p53 [119]. However, p53^4KR98^ (3KR with an additional mutation, K98R) and p53 (P47S, a SNP specific to African populations) failed to inhibit SLC711A expression or tumor growth [123]. Spermidine/spermine N1-acetyltransferase 1 (SAT1), which is critical for cell polyamine catabolism mediated through acetylation, is also a target gene of p53 (Fig. 1) [124]. SAT1 knockdown suppressed p53-induced ferroptosis by specifically affecting the expression of ALOX15, not ALOX5 or ALOX12, to modulate ROS-triggered lipid peroxidation [124]. Pharmacological inhibition of ALOX15 alleviated SAT1-regulated ferroptosis, indicating that the activation of the p53-SAT1-ALOX15 signaling pathway promoted ferroptosis.

p53 also regulates ferroptosis by modulating glutamine metabolism. For example, the level of glutaminase 2 (GSL2), an important mitochondrial glutaminase, is transcriptionally regulated by p53 (Fig. 1) [125]. GLS2 converts glutamine to glutamate, reducing the mitochondrial respiration rate and cellular GSH, ultimately promoting ferroptosis. A recent study demonstrated that the ability of a p53 variant, a nonsynonymous single-nucleotide polymorphism referred to as P47S found in people of African descent, to transactivate GLS2 was impaired [120, 126]. Cancer cells expressing P47S showed lower GLS2 expression levels and were more resistant to ferroptosis. Recent studies have demonstrated that p53 contributes to iron homeostasis by transcriptionally regulating the expression of solute carrier family 25 member 28 (SLC25A28) and ferredoxin reductase (FDXR) to enhance the susceptibility of cells to ferroptosis [127, 128]. Moreover, p53 enhances the entry of iron into cells by mediating the action of lncRNA PVT1 to upregulate the expression of TFR1 [129].

p53 also promotes ferroptosis through vitamin K metabolism. As a recently identified ferroptosis inhibitor, VKORC1L1 is a direct target of p53 (Fig. 1) [116]. Activation of p53 repressed the transcription of VKORC1L1 by binding to the p53-binding sequence in the promoter [116]. VKORC1L1 overexpression significantly attenuated tumor growth suppression in p53 wild-type tumors but not in p53-null tumors, suggesting a new pathway for p53-controlled ferroptosis and tumor growth inhibition mediated by vitamin K metabolism modulation.

#### Anti-ferroptotic roles of p53

A recent study demonstrated that p53 inhibited ferroptosis by regulating the localization and activity but not the expression of dipeptidyl peptidase-4 (DPP4) in a transcription-independent manner (Fig. 1) [130]. DPP4 is a ubiquitous enzyme that activates lipid peroxidation by interacting with NADPH oxidase 1 (NOX1). Mechanistically, p53 forms a complex with DPP4 and promotes the nuclear accumulation of DPP4; thus, plasma membrane-associated DPP4-mediated lipid peroxidation is prevented, and ferroptosis is inhibited (Fig. 1) [130]. Depletion or pharmacological inhibition of p53 potentiated the anticancer activity of erastin and sulfasalazine. In contrast, elevated p53 protein levels mediated by the MDM2 inhibitor nutlin-3 suppressed erastin-induced ferroptosis in some cancer cells. Moreover, p53-regulated CDKN1A/p21 expression attenuated ferroptosis by promoting the generation of cellular GSH (Fig. 1) [131, 132]. CDKN1A/p21 is an inhibitor of cyclin-dependent kinase (CDK) and the first identified target gene of p53. CDKN1A/p21 regulates cell cycle arrest by inhibiting the formation of CDK complexes, including cyclin E/A-CDK2 and cyclin D-CDK4/6. However, CDK4/6 inhibitors do not suppress ferroptosis, suggesting that CDKN1A/p21 inhibits ferroptotic cell death through other unknown signaling pathways [131, 132]. In addition, the calcium-independent phospholipase iPLA2β has been identified as a target gene of p53 that suppresses ferroptosis by detoxifying peroxidized lipids under mild stress conditions (Fig. 1) [133]. Inhibition of iPLA2β significantly elevated the sensitivity of cells to p53-driven ferroptosis in vitro and in vivo. However, when the stress level exceeded a threshold, p53 promoted the activation of ferroptosis instead of activating iPLA2β [133]. These results emphasize the dual roles of p53 in ferroptosis.

Despite the increase in understanding the roles of p53, the molecular switch between p53-regulated ferroptosis and other types of cell death, such as apoptosis, is still poorly understood. A better understanding of the precise molecular mechanisms by which p53 mediates ferroptosis in cancer cells and different organ cells will guide the development of new treatments for human diseases.

## The relationship between ferroptosis and kidney disease

Increasing evidence suggests that ferroptosis is widely involved in the pathological process of a variety of human diseases, including heart diseases, neurodegenerative diseases, cancer and multiple kidney diseases. It is important to explore the roles and mechanisms of ferroptosis in kidney disease, as targeting ferroptosis is important for the prevention and treatment of kidney diseases. Here, we systematically elaborate on the link between ferroptosis and kidney diseases and provide more possibilities and information for the treatment and prevention of kidney diseases.

### Ferroptosis and acute kidney injury (AKI)

Ischemia‒reperfusion injury (IRI), rhabdomyolysis, and chemical drugs are common causes of AKI [134, 135]. In folic acid (FA)-induced AKI, Z-VAD-FMK, an inhibitor of apoptosis, could not reduce the damage to renal tubular epithelial cells, while the ferroptosis inhibitor Fer-1 effectively reduced oxidative stress and renal tubular cell death [136]. In a mouse model of AKI induced by rhabdomyolysis, Fer-1 blocked cell death by inhibiting lipid peroxidation, again revealing the association between ferroptosis and AKI [137]. Cumulative studies have demonstrated that iron chelators and small molecule inhibitors of ferroptosis have protective effects in various AKI animal models [138, 139]. Therefore, ferroptosis has become one of the therapeutic targets of AKI. The application of inhibitors of ferroptosis has become a new strategy for the treatment and prevention of AKI.

#### Ferroptosis and renal IRI-induced AKI

IRI is defined as pathological cell damage induced by blood reperfusion into the organ suffering from ischemic injury [140]. In clinical practice, IRI can lead to severe AKI and delayed functional recovery after organ transplantation [141]. IRI is an important inducer of AKI [142]. IRI consists of two stages: the first stage is hypoxia-ischemia, which is characterized by energy failure and cell death primarily caused by apoptosis; in the reperfusion stage, ROS are overproduced, and ferroptosis is induced [143, 144]. Moreover, autophagy occurs in human renal tubular epithelial cells during both pathological processes [145, 146]. In summary, the main mechanisms of ferroptosis during IRI are excessive ROS production, cascade-amplified inflammatory reactions and ferritinophagy.

**Fig. 2:**
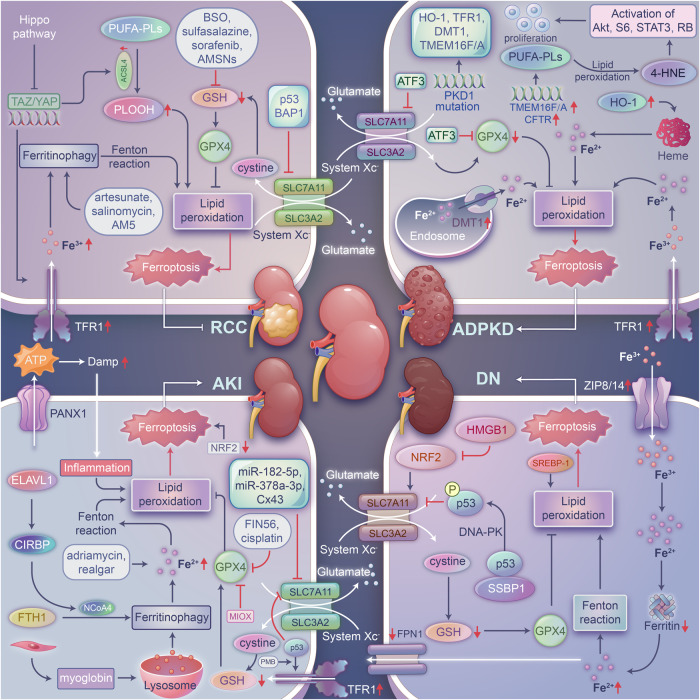
The regulatory roles of ferroptosis in various kidney diseases. Many ferroptosis-associated genes contribute to the development of RCC, ADPKD, AKI, and DN. Ferroptosis inhibitors or activators exhibit protective effects on different kidney diseases.

In the mouse IRI model, the expression of GPX4 and SLC7A11, two key protective genes for ferroptosis, was significantly decreased compared with that in the control group [147]. The noncoding RNAs miR-182-5p and miR-378a-3p bound directly to the 3’ UTRs of GPX4 and SLC7A11 mRNA and negatively regulated their expression. Therefore, inhibition of miR-182-5p and miR-378a-3p can indirectly inhibit ferroptosis and alleviate IRI-induced renal injury (Fig. 2) [147]. Silencing pannexin 1 (PANX1), an ATP release channel, reduced the expression of proinflammatory molecules, upregulated HO-1 expression, attenuated the MAPK/ERK pathway to alleviate NCOA4-mediated ferritinophagy, and ultimately inhibited ferroptosis and alleviated IRI-AKI (Fig. 2) [148]. Augmenter of liver regeneration (ALR) has been shown to prevent IRI-AKI by modulating system Xc-GSH-GPX signaling and scavenging ROS to inhibit ferroptosis [149, 150]. ACSL4 expression was significantly upregulated in the renal tissues of FA- or IRI-treated mice [151]. Mechanistically, HIF-1α directly bound to the promoter of the ACSL4 gene and regulated ACSL4 transcription (Fig. 1). Knockout of ACSL4 mitigated renal pathological damage in IRI-AKI mice by decreasing the inflammatory response, suppressing immune cell infiltration and suppressing ferroptosis [151]. Consistently, the ACSL4 inhibitor rosiglitazone also had kidney protective effects against IRI-induced AKI by reducing the infiltration of immune cells and ferroptotic cell death [48, 151].

Ferritinophagy facilitates ferroptosis by regulating iron metabolism, and it is involved in many pathophysiological processes, such as oxidative stress and iron overload [34, 152]. Ferritinophagy is enhanced in renal cells undergoing hypoxia/reoxygenation (H/R) and ferroptosis, and downregulation of cold-induced RNA-binding protein (CIRBP) expression prevents ferritinophagy and further renal IRI (Fig. 2) [153]. ELAVL1 is a promoter of ferroptosis that may induce ferroptosis by positively regulating CIRBP expression [153]. Therefore, knockdown of ELAVL1 or CIRBP can inhibit ferritinophagy and thus suppress ferroptosis [153].

#### Ferroptosis and rhabdomyolysis-induced AKI

Rhabdomyolysis (RM) is a life-threatening clinical syndrome. The common complication of RM is AKI, and approximately 10% of AKI patients present with RM. Numerous studies have shown that the accumulation of myoglobin in the kidney is an important cause of kidney damage [154]. Excessive myoglobin enters the lysosomes of tubular cells and is decomposed into globulin and high levels of heme, which are further metabolized to produce a large amount of free iron [155, 156]. Accumulated iron induces lipid peroxidation through the Fenton reaction, causing acute renal tubular injury [134, 157–159]. In addition, FTH1 converts Fe^2+^ to Fe^3+^, which attenuates lipid peroxidation caused by high levels of free iron and inhibits ferroptosis. In a mouse AKI model, knockdown of FTH1 aggravated RM- and cisplatin-induced renal injury, suggesting that FTH1 may play a protective role against ferroptosis in the context of AKI [160, 161]. Interestingly, iron deficiency also aggravated RM-induced AKI by enhancing catalytic heme-iron-modulated lipid peroxidation and DNA oxidation, upregulating the activity of p53/p21 and promoting cellular senescence [162].

#### Ferroptosis and chemical drug-induced AKI

Exposure to chemical drugs is a common risk factor for AKI. Excessive FA caused AKI in both human and animal models. In a mouse model of FA-induced AKI, Fer-1 significantly restored renal function, but apoptosis inhibitors were not effective, suggesting a dominant role for ferroptosis in FA-induced AKI [136, 163]. In the FA-AKI model, FA-induced ferroptosis exacerbated renal injury by triggering inflammation to induce other types of cell death, consistent with the increase in the expression of TWEAK and Fn14 in renal tubular epithelial cells in the AKI context [164–166]. Adriamycin is a type of chemotherapy that is effective against a wide range of human cancers. However, adriamycin-induced cardiac toxicity and nephrotoxicity are the most common side effects that limit its long-term use [167]. Interestingly, typical characteristics of ferroptosis were clearly observed in kidney cells in adriamycin-treated rats; these changes included morphological alterations of mitochondria, iron accumulation, and oxidative stress [168]. Realgar, a Chinese medicine containing arsenic, has been used to treat carbuncles, furuncles, and multiple cancers. However, realgar exposure induced nephrotoxicity in mice by initiating ferroptosis, which was accompanied by iron accumulation, excessive ROS production, and inhibited expression of key ferroptosis regulators. In addition, realgar-induced ferroptosis was suppressed by Fer-1 in HK-2 cells (Fig. 2) [169].

Cisplatin is a nonspecific cytotoxic drug targeting the cell cycle by inhibiting the DNA replication process in cancer cells and damaging the structure of the cell membrane, endowing it with broad spectrum of anticancer efficacy. Cisplatin exerts a strong promoting effective via accumulation, therefore, it tends to induce nephrotoxicity and often causes renal cell damage [170]. Increasing evidence suggests that cisplatin-induced AKI depends on ferroptosis, as cisplatin-treated animals exhibit typical features of ferroptosis. In an cisplatin-induced AKI mouse model, the levels of peroxidation markers in mice with loss of vitamin D receptor (VDR) were much higher than those in wild-type mice, thus exacerbating ferroptosis in mice [171]. In addition, GPX4 has been proven to be a target gene of VDR; therefore, the VDR-GPX4 axis may be an ferroptosis-inhibiting pathway (Fig. 2) [171]. However, overexpression of myo-inositol oxygenase (MIOX) can aggravate cisplatin-induced ferroptosis in the context of AKI by downregulating GPX4 activity, promoting lipid peroxidation and increasing the ferritinophagy rate [172]. Cisplatin treatment upregulated the expression of P66Shc in vivo and in vitro and promoted its mitochondrial translocation to exacerbate ferroptotic cell death and induce cisplatin-induced AKI [173]. In addition, downregulation of Cx43 (also known as gap junction protein alpha 1, GJA1) expression restored the level of SLC7A11, inhibited ferroptosis and ultimately alleviated cisplatin- and LPS-induced AKI [174]. Loss of farnesoid X receptor (FXR) exacerbated the ferroptosis signaling pathway by enhancing iron accumulation, increasing lipid peroxidation, decreasing GSH levels and reducing GPX4 expression in cisplatin-treated mice and HK-2 cells [175]. In contrast, these outcomes were reversed by the FXR agonist GW4064. Furthermore, RNA-sequencing analysis results implied that GW4064 treatment influenced the expression of ferroptosis-related genes, glutathione metabolism-related genes, lipid metabolism-related genes, and oxidative stress-related genes. Chromatin immunoprecipitation-sequencing (ChIP-seq) results also confirmed that FXR bound to the promoters of several ferroptosis-associated genes, including FPS1, GGT6, and GSTA4. GW4064 treatment further elevated the occupancy of FXR on FXR response elements (FXREs) in these ferroptosis regulatory genes. In addition, the FXR-MAFG pathway suppressed the expression of oxidative stress-related genes, such as HO-1, NQO-1, and transferrin (TF). The protective effect of FXR on the kidney is realized by its direct or indirect regulation of the expression of ferroptosis-related genes [175, 176]. Recently, two important genes, DPEP1 and CHMP1A, were identified as regulators of kidney diseases through genome-wide association studies (GWAS). Loss of DPEP1 markedly relieved FA- or cisplatin-induced AKI by altering the apoptosis and ferroptosis, but not the pyroptosis or necroptosis. In contrast, CHMP1A haploinsufficiency greatly exacerbated FA- or cisplatin-induced AKI by promoting ferroptosis through iron accumulation [177]. DPEP1 and CHMP1A regulate the development of AKI by mediating the balance between iron metabolism and ferroptosis.

Ferroptosis plays an important role in polymyxin B (PMB)-induced AKI (Fig. 2) [178]. Notably, p53 was upregulated in PMB-treated mice and HK-2 cells. Silencing p53 significantly alleviated PMB-induced iron accumulation and lipid peroxidation, increased the expression of TFR1 and ALOX12, and decreased the expression of GPX4 and SLC7A11 [178]. Thus, activated p53 promotes ferroptosis in PMB-induced AKI by downregulating SLC7A11 expression and upregulating TfR1 expression.

#### Ferroptosis inhibitors and AKI

Considering that the roles of ferroptosis in AKI have been gradually revealed, there have been many reports on ferroptosis inhibitors for the treatment of AKI. The ferroptosis inhibitors evaluated to date mainly play a role by inhibiting lipid peroxidation, upregulating GPX4 expression, and maintaining iron homeostasis.

##### Specific ferroptosis inhibitors and antioxidants

Fer-1, as a first generation specifically synthesized ferroptosis inhibitor, inhibits ferroptosis induced by RAS selective lethal small molecule 3 (RSL-3) but does not inhibit cell death induced by any other oxidative lethal compound or apoptosis inducer. Moreover, Fer-1 inhibits erastin-induced lipid peroxidation and ferroptotic cell death [2, 179, 180]. Recent in vivo and in vitro experimental evidence suggests that Fer-1 effectively alleviates AKI by inhibiting ferroptosis [136, 181]. Lip-1 is a specific ferroptosis inhibitor that suppresses ferroptosis when administered at low concentrations without interfering with other cell death processes. Lip-1 alleviates ferroptosis in renal tubules and liver tissue after IRI [8, 180, 182]. Vitamin E is one of the most important lipophilic free radical-trapping antioxidants, and its defense mechanism against ferroptosis-induced kidney disease is mainly manifested in patients with AKI. Vitamin E inhibits ferroptosis by preventing the cisplatin-induced decline in the renal antioxidant defense system or by its direct free radical-scavenging activity [183]. In addition, vitamin E deficiency also leads to the aggravation of renal IRI [184]. Recent studies have shown that vitamin K1 protected against IR-induced AKI by targeting the ferroptosis pathway, whereas the vitamin K antagonist phenprocoumon further aggravated the symptoms of AKI in mice. Consistent with these in vivo results, vitamin K1 treatment impeded RSL3- or erastin-induced ferroptosis by not only regulating GPX4 expression but also modulating the levels of FSP1 and DHODH, two newly identified factors that defend against ferroptosis [114]. In addition, the inhibitory effect of vitamin K1 on ferroptosis is mediated among cells, suggesting that vitamin K1 may be effective in the treatment of other ferroptosis-related diseases.

Lipoic acid (LA) reduces cellular iron overload by upregulating the expression of ferritin and FPN1. It also attenuates FA-induced AKI by blocking ferroptosis by increasing the levels of GSH and GPX4 and reducing ROS accumulation and lipid peroxidation rates [185]. CoQ_10_ generated via the mevalonate pathway functions as an endogenous antioxidant, which inhibits ferroptosis by reducing the accumulation of intracellular lipid peroxide [186, 187]. FIN56 induces the posttranslational degradation of GPX4 and the depletion of CoQ_10_, thereby inducing ferroptosis. FIN56-induced ferroptosis is alleviated via supplementation with idebenone and selenite [186, 188]. Visomitin, also known as SKQ1, is a novel mitochondrion-targeting antioxidant. Visomitin exhibited excellent renal protective effects in FA-, cisplatin-, and IR-induced models by reducing lipid peroxidation and mitochondrial ROS generation to ameliorate ferroptosis in kidneys in vivo and in vitro [189]. Thus, blocking ferroptosis by targeting mitochondria may be a prospective therapeutic direction in AKI.

##### Natural polyphenolic compounds

Quercetin is a natural bioflavonoid compound ubiquitous in fruits and vegetables and shows potential therapeutic use in various human diseases. Quercetin is a potent inhibitor of ferroptosis. It inhibits ferroptosis by inhibiting the expression of activating transcription factor 3 (ATF3), reducing the levels of lipid ROS and increasing the levels of GSH, which ultimately attenuates IRI- or FA-induced AKI [190]. Isoliquiritigenin (ISL), one of the most important chalcone compounds derived from licorice root, exhibits multiple bioactivities. ISL attenuates septic AKI by inhibiting Fe^2+^ and lipid peroxide accumulation and inhibiting ferritinophagy [191]. Baicalein is a flavonoid extracted from the roots of *Scutellaria baicalensis* and *Scutellaria lateriflora* that exhibits a variety of biological processes, showing antioxidant and anti-inflammatory properties. As a natural ferroptosis inhibitor, baicalein regulates iron homeostasis and inhibits the Fenton reaction, lipid peroxidation, and erastin-induced degradation of GPX4 [192–195]. Curcumin is a natural polyphenol that shows great potential for use in the treatment of human diseases. The antioxidant curcumin alleviates Mb-mediated inflammation and oxidative stress by inhibiting the TLR4/NF-κB axis and activating NRF2-induced HO-1 expression; therefore, curcumin may play a role in renal protection (Fig. 2) [99, 196, 197]. Gastrodin alleviates glutamate-induced ferroptosis through the NRF2/HO-1 signaling pathway [198].

##### Iron chelators

Deferoxamine (DFO), ciclopirox olamine (CPX), 2,2-bipyridyl and other iron chelators can directly bind free iron, maintain the balance of iron metabolism in vivo and inhibit ferroptosis induced by erastin (Fig. 2) [2, 199–201]. Moreover, studies have shown that DFO inhibited lipid peroxidation and alleviated AM-AKI [202]. Deferiprone (DFP), an oral alternative to DFO, is often used in clinical practice [179, 203]. The protective effect of DFP on the kidneys needs to be further investigated, and researchers may consider how to apply it to clinical practice.

##### NRF2 activators

According to the aforementioned research, NRF2 is abundant in the kidneys and plays a central role in maintaining redox status by regulating genes encoding antioxidant and detoxifying molecules. The activity and expression of NRF2 were decreased in many AKI and CKD animal models, suggesting that NRF2 was an important mediator in the pathogenesis and progression of AKI and CKD [204, 205]. The nephroprotective effect of NRF2 is supported by the fact that loss of NRF2 in mice exacerbated diabetes-induced oxidative stress, inflammation, and kidney damage [206]. Moreover, compared to wild-type mice, IRI or nephrotoxic insults led to more severe kidney injury and renal dysfunction in NRF2-deficient mice, as well as higher mortality [207]. Consistent with these observations, many NRF2 activators found in foods or dietary supplements exhibit nephroprotective effects in various animal models (Fig. 1). These findings highlight that targeting NRF2 provides a novel therapeutic approach for preventing and treating human kidney diseases.

Lentinus edodes polysaccharide induces the expression of NRF2 and promotes its binding to antioxidant reactive elements (AREs), which elevates the expression of downstream antioxidant genes and alleviates cisplatin-induced and ROS-mediated nephrotoxicity [27, 208]. Pseudoginsenoside reverses the reduction in deacetylase 1 expression induced by cisplatin and indirectly activates NRF2 to alleviate ferroptosis [209]. Dioscin, a type of steroidal saponin, protects against cisplatin-induced AKI by suppressing ferroptotic cell death through its activation of the NRF2-HO1 pathway. Inhibition of NRF2 dramatically reduced the nephroprotective effect of dioscin in the context of AKI [210]. Poria acid (PA) upregulated the expression of GPX4, SLC7A11, and HO-1 by increasing the level of GSH and activating NRF2 in a mouse IRI-AKI model, which inhibited the induction of ferroptosis and attenuated AKI [8, 110]. Formononetin and farrerol activate the Keap1-NRF2 signaling pathway and attenuate cisplatin-induced AKI [211, 212]. In addition, natural activators of NRF2, such as indole, diterpenes, coumarin and lactone, alleviate renal cell death induced by lipid peroxidation [213]. Entacapone is a specific peripheral catechol-*O*-methyltransferase inhibitor approved by the US FDA in 1999 to treat patients with advanced Parkinson’s disease as an adjuvant drug. Entacapone also exhibited anti-ferroptosis activity to alleviate IRI-induced renal damage and pathological changes in mice and erastin- or RLS3-induced lipid peroxidation and iron accumulation. Moreover, entacapone activated the p62-Keap1-NRF2 signaling pathway to increase the expression of SLC7A11 and reduce oxidative stress [214].

##### Alkaloids

Nuciferine, an alkaloid extracted from lotus leaves, can directly inhibit ferroptosis by reducing cellular oxidative stress, reducing iron accumulation and preventing lipid peroxidation, thereby preventing FA-induced acute kidney injury [166]. Notably, the renoprotective effect of nuciferine was dependent on GPX4 [166]. Leonurine, an important alkaloid isolated from *L. sibiricus*, greatly inhibited lipid peroxidation and iron accumulation in cisplatin-induced AKI contexts by activating NRF2 [215].

##### Other inhibitors

Paricalcitol restores GPX4 expression by activating VDR, improves renal function, reduces mitochondrial damage, and alleviates cisplatin-induced AKI [163, 171, 179]. Serum irisin levels were reduced in I/R-induced AKI mice. Irisin treatment can alleviate renal injury, reduce the inflammatory response, increase mitochondrial function, and mitigate oxidative stress after IR injury, and its effects are accompanied by the upregulation of GPX4 expression and inhibition of ferroptosis (Fig. 2) [216]. Consistently, irisin treatment attenuated sepsis-related AKI induced by cecal ligation and puncture (CLP) not only by blocking the ferroptosis signaling pathway but also by upregulating SIRT1/NRF2 axis activation [217]. Notably, the protective effects of irisin were abated by the administration of the SIRT1 inhibitor EX527 in vivo or by siRNA-mediated knockdown of SIRT1 in vitro.

Ruscogenin, a major bioactive steroidal sapogenin, is derived from the traditional Chinese herb *Ophiopogon japonicus*. Ruscogenin treatment significantly relieved FA-induced AKI, improved kidney function indicators, restored the expression of SLC7A11 and HO-1 and suppressed the FA-induced upregulation of Rev-erbα/β. In summary, ruscogenin alleviated AKI through the Rev-erbα/β-SLC7A11/HO-1 signaling pathway to block ferroptosis [218]. Ginsenosides constitute a class of glycosylated triterpenes, also known as saponins, which are the major bioactive constituents of ginseng root. Ginsenoside Rg1 is a major ginsenoside in ginseng. Ginsenoside Rg1 treatment ameliorated sepsis-induced AKI by reducing iron deposition and lipid peroxidation and elevating the expression of GPX4 and FSP1. The anti-ferroptosis effect of ginsenoside Rg1 seems to be dependent on FSP1 because knockdown of FPS1 greatly impaired the protective effect on LPS-treated cells [219]. Astragaloside IV, a major compound extracted from the aqueous extract of *Astragalus membranaceus*, shows broad application prospects, especially in the heart, kidney, liver, lung, endocrine system, nervous system and immune system. More importantly, Astragaloside IV markedly alleviated kidney dysfunction, decreased oxidative stress, and ameliorated iron deposition to prevent ferroptosis. In addition, Astragaloside IV treatment increased the phosphorylation of PI3K and Akt to promote the restoration of NRF2 expression and nuclear translocation [220]. Moreover, carbenoxolone inhibited PANX1 activity and attenuated IR injury to protect the kidney [221]. Thiazolidinedione (TZD) compounds, such as rosiglitazone (ROSI), pioglitazone (PIO), and troglitazone (TRO), reduced the mortality of mice after GPX4 was loss by inhibiting the action of ACSL4, indicating that these compounds may show potential therapeutic effects on AKI [43, 48]. Other inhibitors, such as XJB-5-131, dibutyl-hydroxytoluene, and tert-butyl-hydroxyanisole, also inhibit lipid peroxidation and relieve oxidative stress to block ferroptosis, thereby protecting the kidney against various injuries [222].

### Ferroptosis and chronic kidney disease (CKD)

CKD refers to renal structural and functional abnormalities induced by various factors lasts more than 3 months, and includes a variety of kidney disease types, among which diabetic nephropathy (DN) and polycystic nephropathy are the most closely related to ferroptosis. CKD is caused by many heterogeneous diseases that are mediated through different pathways, and CKD induces irreversible changes and persistent damage to renal function and structure within months or years. The final pathological manifestation of CKD is renal fibrosis, characterized by glomerulosclerosis, renal tubular atrophy, and interstitial fibrosis [223, 224]. An increasing number of studies have proven that ferroptosis is closely related to CKD. Iron overload is evident in many CKD models, such as ZSF1, diabetic db/db, and streptozotocin (STZ) models [225]. In the ZSF1 rat model, the renal iron concentration is significantly increased, and serum creatinine and urinary protein levels are positively correlated with the level of renal iron. Elevated renal iron levels promote ALOX activation and oxidative stress. Iron deposition and ALOX are the main mechanisms underlying lipid peroxidation [226]. In the db/db diabetic mouse model, mice in the low-iron diet group showed lower urinary protein excretion, renal MDA levels, and oxidative stress than those in the normal diet group [227].

Characteristic mitochondrial morphological changes in ferroptosis are observed in cells cultured with high glucose [228]. In STZ-induced DN mouse kidney sections, the outer mitochondrial membrane ruptured, mitochondrial crista disappeared, ACSL4 expression was significantly increased, the levels of oxidative products were elevated, and the level of GPX4 was decreased, indicating that ferroptosis was involved in STZ-induced DN [225]. In the kidneys of STZ-induced diabetic mice and HK-2 cells cultured with high glucose, iron overload, decreased antioxidant capacity and high rates of ROS production and lipid peroxidation are the signature changes of ferroptosis [225, 228].

#### Ferroptosis and diabetic nephropathy (DN)

DN is a common microvascular complication of diabetes mellitus and the main cause of end-stage renal disease (ESD). The pathogenesis of DN includes abnormal glucose metabolism, hyperperfusion and hyperfiltration of the kidney, oxidative stress, and inflammation. Recent studies suggest that ferroptosis is involved in the pathogenesis and progression of DN. DN related to ferroptosis mainly manifests as oxidative stress, lipid peroxidation, and iron homeostasis disorders [229, 230]. Among these symptoms, lipid peroxidation is evident throughout the progression of DN, and the levels of the products of lipid peroxidation, namely, MDA and 8-iso-PGF2α can be indicators to judge or predict the degree of kidney injury in the early stage of DN. High-fat diet (HFD)-induced diabetic mice exhibited more severe renal damage, including higher renal injury scores, elevated levels of serum BUN (blood urea nitrogen), CCr (creatinine clearance rate) and Cysc (cystatin C), oxidative stress and ferroptosis.

In the pathological progression of DN, iron deposition leads to ferroptosis and destroys kidney cells. Exposure of renal tubular epithelial cells to increased iron leads to renal cell damage because free radicals are generated through the Fenton reaction. In the context of CKD, iron deposition is accompanied by the increased expression of iron input proteins (ZRT/IRT-like proteins, ZIP14 and ZIP8) or the decrease in FPN1 expression, indicating that iron accumulation may be triggered by increased iron uptake or insufficient iron efflux (Fig. 2) [231]. ZIP14 is a member of the SLC39A transporter family, which regulates the cellular uptake of metal ions, such as zinc, iron, and manganese. ZIP14 is upregulated during ferroptosis in rats with STZ-induced DN. Overexpression of ZIP14 results in an increase in intracellular iron levels, which leads to disordered iron homeostasis and ferroptosis [232]. Therefore, regulating the expression of iron metabolism-associated proteins is of great importance for restoring iron homeostasis and reducing ferroptosis in the kidney.

In addition, oxidative stress and inflammation related to ferroptosis lead to kidney cell damage and DN. Antioxidants, including that of the antioxidants SOD, CAT, and GSH-Px, effectively inhibit ferroptosis in DN. In DN cells, the use of Fer-1 is beneficial because it increases the expression of antioxidative genes to maintain redox homeostasis and alleviate ferroptosis [228]. NAC, an antioxidant, blocks high glucose (HG)-induced ferroptosis by enhancing mitochondrial GSH activity and upregulating GPX4 expression. NAC also maintains mitochondrial redox homeostasis by activating the SIRT3-SOD2-GPX4 signaling pathway, thus reducing the ferroptosis rate in the context of DN. NAC treatment reduces the upregulated SOD2 acetylation level [233]. In addition, HO-1 is specifically expressed in the glomeruli of the context of DN, and the induction of HO-1 prevents podocyte apoptosis [234, 235]. Consistent with these conclusions, the expression of ferroptosis-related factors in DN cells is unbalanced, accompanied by a significant increase in the HGMB1 level. Knockout of HGMB1 promoted the expression of NRF2 and its downstream targets, including HO-1, NQO1, GCLC, and GCLM, prevented the production of ROS and LDH and upregulated the level of GPX4 in renal mesangial cells (Fig. 2) [236]. All these results indicated that HMGB1 regulates oxidative stress induced by ferroptosis when exposed to high levels of glucose and that DN caused by ferroptosis can be alleviated by knocking out the HMGB1 gene. In addition, sterol regulatory element-binding proteins (SREBPs), such as SREBP-1, in diabetic kidneys aggravated DN by increasing lipid synthesis [237]. Specific protein 1 (SP1)-mediated upregulation of PRDX6 expression also prevented iron overload by regulating iron metabolism, restoring SLC7A11 activity and GSH activity, and further promoting the increase in GPX4 expression to prevent ferroptosis-associated DN [238]. Recently, an interesting study revealed that fructose at a high level triggered progressive glomerular injury by initiating ferroptosis and reported that mitochondrial single-strand DNA-binding protein 1 (SSBP1) is an important regulator in the glomeruli of high fructose-fed rats, as determined via quantitative proteomic analysis. Mechanistically, SSBP1 interacted with p53 and promoted DNA-PK-dependent phosphorylation of p53 at S15 to facilitate the nuclear accumulation of p53, which ultimately inhibited the transcription of SLC7A11 during ferroptosis in high fructose-treated glomerular podocytes (Fig. 2). Consistent with these observations, treatment with pterostilbene, a natural inhibitor of SSBP1, effectively suppressed DNA-PK/p53 axis activation to alleviate high fructose-triggered ferroptosis in the glomerular podocytes. Therefore, the SSBP1-DNA-PK-p53-SLC7A11 signaling pathway is closely associated with high fructose-induced podocyte ferroptosis, and inhibition of SSBP1 may be a new therapeutic approach [239, 240]. Our previous studies showed that SSBP1 also interacted with heat shock factor 1 (HSF1) to mediate the mitochondrial unfolded protein response by regulating mitochondrial chaperone levels [241]. HSF1 is a conserved transcription factor that coordinates the heat shock response by transcriptionally regulating the expression of heat shock proteins (HSPs). In addition, HSF1 has also been reported to be critically involved in ferroptosis by inducing HSPB1 expression [28]. Therefore, it will be interesting to explore whether the HSF1-SSBP1 complex modulates ferroptosis and kidney diseases and to identify new targets of HSF1 that may be closely associated with ferroptosis.

Based on the aforementioned research, ferroptosis inhibitors have been shown to exert certain therapeutic effects on DN. Fer-1 treatment lowered the expression of ZIP14 and decreased the levels of cellular iron and MDA, which was consistent with improved kidney function in DN rats [232]. Fer-1 treatment effectively suppressed ferroptosis in LPS-induced septic AKI mice fed a HFD. In addition, Fer-1 alleviated DN and ameliorated renal hypertrophy and albuminuria by inhibiting ferroptosis, and ultimately reduced the accumulation of intracranial lipid peroxides in diabetic mice through the HIF-1α/HO-1 pathway [242]. During this process, NADPH oxidase was activated and upregulated. Notably, treatment with Vas2870, an inhibitor of NADPH oxidase, significantly increased the survival rate of HFD-fed mice subjected to LPS by ameliorating renal injury and inhibiting ferroptosis. Therefore, targeting NADPH-regulated release of ROS and ferroptosis may be a novel therapeutic strategy to treat DN [243]. Rosiglitazone, an inhibitor of ACSL4, reduced the contents of MDA and iron in the kidneys of DN mice, thus increasing the survival rate and renal function of these mice [225]. Dapagliflozin, which functions primarily by blocking glucose reabsorption in the proximal tubule by targeting sodium-glucose cotransporter 2 (SGLT2), is a hypoglycemic agent used in the clinic to treat diabetes. Dapagliflozin treatment also alleviated tubular injury in diabetic model mice by inhibiting ferroptosis (Fig. 2). Interestingly, dapagliflozin may bind with FPN1 to regulate the ubiquitination and degradation of FPN1 [244]. Calycosin is an isoflavone and a natural phytoestrogen extracted from Astragali Radix. Calycosin has many pharmaceutical properties and has a long clinical history of use in the treatment of human diseases, including DN. A recent study showed that calycosin elicited its beneficial effects through its inhibition on ferroptosis, which was realized by controlling lipid ROS production and iron import in high glucose-treated HK-2 cells and db/db mice [245]. Schisandrin A, a bioactive lignan isolated from the traditional Chinese medicine Fructus schisandrae chinensis, significantly alleviated high glucose-promoted ferroptosis and ROS-triggered pyroptosis in a mouse model of DN. Schisandrin A directly interacts with adipoR1 to inhibit its ubiquitination and activate the AdipoR1/AMPK axis. Knockdown of AdipoR1 decreased the protective effects of schisandrin A in a DN mouse model, while the AdipoR1 agonist gramine exerts the opposite effect, indicating that AdipoR1 may be a potential target of SA in a DN model [246]. Glabridin is an isoflavone class of natural phenols isolated from the root of Glycyrrhiza glabra. Glabridin shows a variety of biological activities and therapeutic effects in humans with certain diseases. Glabridin exhibited therapeutic potential in the treatment of DN through improving renal function by repressing not only ferroptosis but also VEGF/p-Akt/p-ERK1/2 axis activation [247]. Nobiletin is a polymethoxylated flavonoid isolated from citrus fruits that exhibits a wide range of physiological effects. A recent study demonstrated that oral administration of nobiletin attenuated pathological alterations, renal fibrosis, leukocyte cell infiltration, and oxidative stress injury in a mouse CKD model established via unilateral ureteral obstruction (UUO). In addition, nobiletin treatment restored the reduced expression of some ferroptosis-related factors, including GPX4, SLC7A11, and TFR1 [248]. Empagliflozin, an inhibitor of sodium-glucose cotransporter 2 (SGL2) that is used to lower blood sugar levels in type-2 diabetes patients, attenuates diabetic kidney damage by reducing ferroptosis through its effect on the AMPK/NRF2 axis [249]. In addition, the inhibition of AMPK action impaired the protective effect of empagliflozin on ferroptosis in the renal cells of DKD mice [249]. The expression of the enzyme cyclooxygenase-2 (COX2) was markedly upregulated in diabetic kidney tissues. Silencing COX2 significantly ameliorated disorders, as indicated by the renal physiological index, and renal tubule injury in diabetic mice [250]. Interestingly, aspirin inhibited the progression of DKD by downregulating COX2 expression to disrupt the ferroptosis pathway. Thus, COX2 may be a potential target of ferroptosis and DKD [250].

#### Ferroptosis and polycystic kidney disease

Autosomal dominant polycystic kidney disease (ADPKD) is one of the causes of end-stage renal disease and is caused by a polycystic protein-1 (PKD1) or PKD2 gene mutation. PKD gene mutation not only leads to ADPKD occurrence but also promotes the development of progressive renal cysts and renal failure [251]. Polycystic kidney disease is characterized by multiple fluid cysts of different sizes in both kidneys; the size of these cysts is gradually increased, eventually distorting the shape and abrogating the function of the kidneys.

Recent studies have demonstrated that the occurrence of polycystic kidney disease is related to ferroptosis. The manifestations closely associated with polycystic kidney disease and ferroptosis include a decrease in GPX activity, imbalanced CFTR channel activity and ROS accumulation, which leads to aggravated lipid peroxidation. Renal cells in the context of ADPKD exhibit a wide range of metabolic abnormalities, including the decreased expression of system Xc- and reduced activity of GPX4, which are the necessary conditions and typical characteristics of ferroptosis [252, 253]. Moreover, the expression of iron import factors (TFR1 and DMT1) and HO-1 is increased (Fig. 2), which leads to a further increase in intracellular iron levels, a decrease in GSH and GPX4 activities, an increase in lipid peroxidation and a tendency to drive ferroptosis [254]. Erastin and Fer-1 could enhance and inhibit ferroptosis and proliferation of PKD1-deficient renal cells, respectively. In PKD1-deficient cells, 4-HNE, as a signaling molecule, promotes cell proliferation and aggravates cyst growth by activating AKT, S6, STAT3, and RB (Fig. 2) [253, 255]. There may be a vicious cycle established in which 4-HNE production promotes GPX4 downregulation and lipid peroxidation, thereby facilitating cyst formation. TMEM16F/TMEM16A function synergistically with the cystic fibrosis transduction regulator CFTR on membrane lipids, induce ROS production and further aggravate lipid peroxidation-mediated ferroptosis (Fig. 2). These results suggest that inhibiting the expression of TMEM16A to suppress lipid peroxidation may be a new therapeutic strategy for ADPKD [252]. In addition, recent studies have indicated that inhibiting or alleviating inflammation by reducing macrophages and inhibiting inflammatory factors can reduce the cyst burden and improve kidney function.

For polycystic kidney disease, several ferroptosis-related therapeutic drugs have been validated in animal models. Fer-1 inhibited ferroptosis and delayed cyst growth in PKD1-mutant mice. Idebenone also exerted a certain inhibitory effect on cyst growth.

#### Ferroptosis and hypertensive nephropathy

Elevated blood pressure is significantly associated with progression of CKD [256]. A high-salt diet not only elevates blood pressure but also increases the serum creatinine (Scr) and BUN levels and promotes inflammation in mice [257]. In an animal model of high-salt diet-induced hypertensive nephropathy, iron accumulation was observed in injured kidneys, accompanied by renal inflammation, mitochondrial dysfunction, oxidative stress, massive proteinuria, and sustained intravascular hemolysis [257, 258]. The ferroptosis inhibitor Fer-1 markedly reduced the blood pressure that had been elevated in the high-salt diet-treated mice [259]. The expression of HO-1, a cytoprotective enzyme critical for heme protein metabolism and ferroptosis, was also highly expressed in iron-accumulating renal cells and in mice with hypertensive nephropathy [257, 260, 261]. High levels of HO-1 in proximal tubular epithelial cells have been significantly associated with proteinuria, hematuria, and tubulointerstitial disease [257, 260, 261]. Previous studies have demonstrated that deletion of HO-1 promoted ferroptosis in renal epithelial cells, suggesting its renoprotective effects in experimental models of kidney diseases [109]. More importantly, the iron chelator DFO significantly alleviated renal damage by preventing iron accumulation, alleviating oxidative stress, inhibiting the inflammatory response, and restoring mitochondrial function [257]. Thus, pharmacological strategies targeting iron homeostatic factors may be effective in treating hypertension-related renal diseases. In addition, in an HHcy-induced 2-kidney, 1-clip (2K1C) hypertensive murine model and Hcy-B CM-treated glomerular endothelial cells (GECs), a significant increase in the ferropotosis rate was triggered through iron accumulation, lipid peroxidation, upregulation of TFR1, and downregulation of SLC7A11 and GPX4 [262]. Mechanistically, HHcy promoted the activation of B cells, which secreted anti-beta 2 glycoprotein I (β2GPI) antibodies that target GECs to increase the number of oxidized phospholipids, thus facilitating lipid peroxidation by activating PE synthases ethanolamine kinase 2 (ETNK2) and ethanolamine-phosphatecytidylyltransferase 2 (PCYT2) [262]. In addition, inhibition of ferroptosis by Fer-1 or pharmacological depletion of B cells effectively alleviated HHcy-induced glomerulosclerosis and hypertensive renal damage [262]. Sirtuin 7 (SIRT7) plays an important role in angiotensin (Ang) II-induced hypertensive renal injury. Ang II pretreatment resulted in high blood pressure, downregulation of SIRT7, and excessive ROS generation, lipid peroxidation and renal ferroptosis by decreasing the expression of GPX4, SLC7A11, and NRF2 [263]. Reduced expression of SIRT7 and increased ferroptotic cell death were also observed in Ang II-treated mouse primary renal tubular epithelial cells (TECs) [263]. Notably, administration of Fer-1 or overexpression of SIRT7 effectively alleviated Ang II-induced epithelial-mesenchymal transition (EMT) and renal ferroptosis in hypertensive mice by suppressing the KIM-1/NOX4 signaling pathway and activating the KLF15/NRF2 and xCT/GPX4 signaling pathways, respectively [263]. These findings indicate that SIRT7 plays an important protective role in ferroptosis and kidney dysfunction in hypertensive renal diseases.

A recent bioinformatics study on the GSE37455 dataset identified three core ferroptosis-related genes, namely, albumin (ALB), nicotinamide N-methyltransferase (NNMT), and ATF3, that were differentially expressed in hypertensive nephropathy samples compared with normal samples [264]. However, the precise roles of these three candidates in hypertensive nephropathy are not fully understood.

In summary, inhibition of ferroptosis may become an effective therapeutic strategy for hypertensive nephropathy. For example, the traditional Chinese medicine Taohongsiwu decoction (THSWD), a multiherb formula, exhibits therapeutic potential in treating several cardiovascular and cerebrovascular diseases. Recently, THSWD has been reported to ameliorate high-salt diet-induced hypertensive nephropathy by suppressing ferroptosis through the p53-NRF2-p21 signaling pathway [259]. Further experimental and human observational studies are needed to clarify and confirm the effects of anti-ferroptotic agents on hypertensive nephropathy.

#### Ferroptosis and IgA nephropathy

IgA nephropathy (IgAN), an immune-mediated chronic kidney disease, is the most common primary glomerulonephritis [265]. Previous studies have demonstrated that the relationship between ferroptosis and iron metabolism is mutually reinforcing and complementary. IgAN induces the dysregulation of iron metabolism, and disturbed iron homeostasis aggravates IgAN progression [266, 267]. A recent study demonstrated that approximately 40% of IgAN patients presented with iron deposition in renal tissues [266]. Interestingly, serum IgA levels were higher in iron-positive IgAN patients than in iron-negative IgAN patients. The levels of urinary protein excretion (UPE), Scr, BUN, and N-acetylb-Dglucosaminidase (u-NAG) were elevated in IgAN patients with a high levels of iron deposition [266]. These findings reveal that the amount of iron deposited in renal tissues is closely correlated with the progression of IgAN and may be an early predictor of IgAN patients.

TFR1 mediates iron homeostasis by regulating iron uptake through the endocytosis of iron-loaded TF. Urinary TF has been positively linked with endothelial cell hyperplasia, mesangial cell hyperplasia, tubular atrophy or interstitial fibrosis, according to the Oxford classification of IgAN patients [267]. Recent studies have suggested that TFR1 functions as a specific ferroptosis marker and plays an essential role in ferroptosis [268]. Interestingly, TFR1 had been previously identified as the major cell surface receptor for IgA1 binding in renal mesangial cells and its overexpression has been correlated with the proliferation of mesangial cells in IgAN patients [269–271]. Human mesangial cells (HMCs) treated with sera from IgAN patients exhibited upregulated expression levels of TFR1 and some inflammatory markers. A recent study demonstrated that IgA1 interacts with its receptor CD89 on mononuclear cells, which releases soluble CD89 (sCD89), and forms IgA1-CD89 complexes [272]. Moreover, IgA1-CD89 complexes promoted the binding of sCD89 with TFR1 to induce the expression of transglutaminase 2 (TGase2) on the surface of HMCs, thereby facilitating the upregulation of TFR1 [272]. The levels of soluble transferrin receptor (sTFR), a fragment of TFR1 on the cell membrane secreted into the circulation, were obviously higher in the blood and urine of IgAN patients than in normal individuals [267, 273]. Moreover, urinary sTFR levels were markedly decreased when IgAN patients were in complete remission [270]. Therefore, TFR1 in blood and urine may be a sensitive indicator for the early diagnosis of IgAN. However, the relationship between TFR1 expression in the glomerular mesangial region and iron dysmetabolism in mesangial cells is still not fully understood, and the reason for the close association of TFR1 with ferroptosis in IgAN patients still needs to be explored.

A recent comprehensive bioinformatics analysis combined with a weighted gene correlation network analysis based on three independent GEO datasets showed that the FABP1 and PPARα-related signaling pathways were involved in IgAN pathogenesis [274]. The expression of FABP1 and PPARα was decreased in IgAN patients compared with control individuals. Galactosedeficient IgA1 (Gd-IgA1) treatment induced ferroptosis in HMCs, and this effect was accompanied by decreased expression of FABP1 and PPARα [274]. Notably, GPX4 expression was also significantly reduced in IgAN renal tissues and Gd-IgA1-treated HMCs in vitro. Moreover, overexpression of PPARα markedly upregulated GPX4 expression and downregulated ACSL4 expression in HMCs [274]. In contrast, knockdown of PPARα led to the opposite effects. These results suggested that the PPARα-FABP1-GPX4 axis may influence the occurrence of ferroptosis to regulate the pathogenesis of IgAN.

More importantly, the levels of MDA were significantly elevated, and the activity of SOD and vitamin E was reduced in the serum of IgAN patients compared with healthy controls [275, 276]. The antioxidant vitamin E (α-tocopherol) has been demonstrated to suppress ferroptosis via LOX suppression [277]. IgAN patients also present with increased concentrations of oxidized free cysteine in plasma, suggesting that the redox balance is disrupted in IgAN patients and that the oxidized cysteine level may be a useful prognostic risk marker for IgAN patients [276]. According to the findings related to the three aforementioned metabolic pathways, in addition to those showing iron accumulation, lipid peroxidation, and redox stress, it is not difficult to infer that ferroptosis plays a vital role in the pathogenesis and progression of IgAN. Thus, ferroptosis inhibitors and iron chelators show therapeutic potential in IgAN treatment.

#### Ferroptosis and nephrolithiasis

Ferroptosis is involved in the development of nephrolithiasis and urolithiasis, as evidenced by the fact that the ferroptosis rate is relatively high in patients with nephrolithiasis and hyperoxaluric mice [278, 279]. Calcium oxalate (CaOx) crystal treatment obviously elevated the cellular iron concentration, enhanced lipid peroxidation, and reduced the expression of GPX4 and SCL7A11 in vivo and in vitro. Fer-1 significantly ameliorated the CaOx-induced renal tubular epithelial cell injury by blocking ferroptosis [278]. More importantly, the p53 expression level and p53 deacetylation rate were significantly increased in patients with nephrolithiasis and in CaOx crystal-treated HK-2 cells, as determined by analyzing the single-cell sequencing data and RNA-sequence data [279]. Moreover, deacetylation of p53 by SIRT1 or via the introduction of three mutations into p53 led to the profound suppression of ferroptosis and alleviated CaOx crystal-induced renal damage [279]. This study revealed the function of p53 in renal fibrosis induced by different CaOx crystals and suggested that targeting the p53 deacetylation pathway may be a potential treatment for patients with nephrolithiasis.

### Ferroptosis and renal cell carcinoma (RCC)

**Fig. 3:**
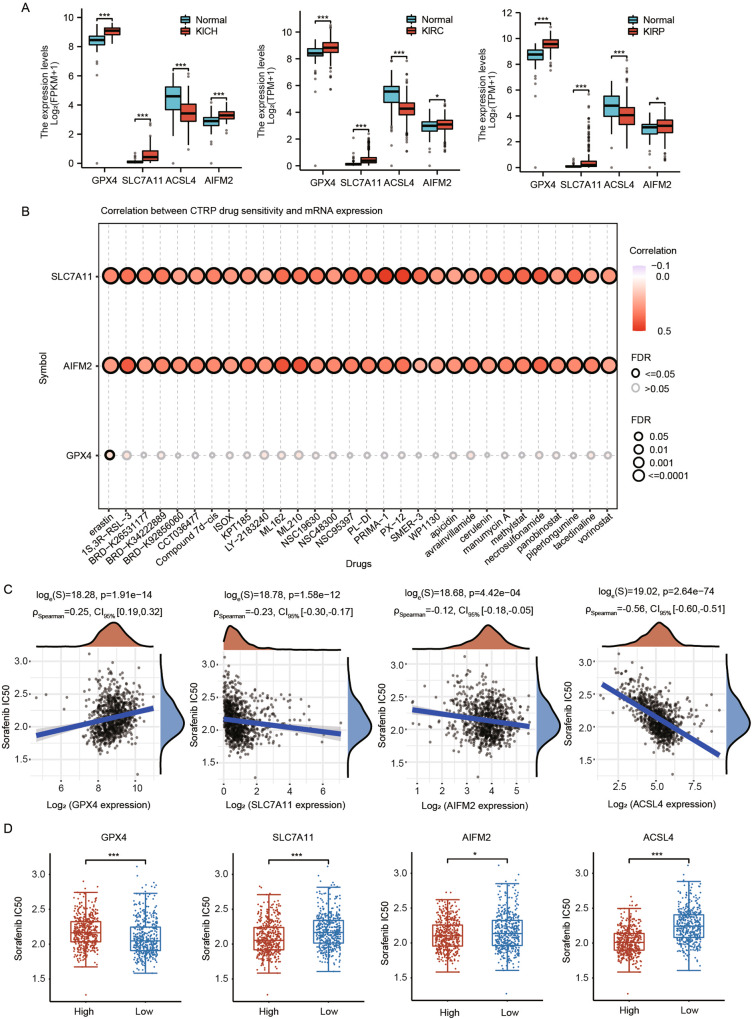
Expression and drug sensitivity of core ferroptosis-related genes in the context of RCC. **A** Expression of GPX4, SLC7A11, ACSL4, and AIFM2 in different kidney cancer tissues and normal tissues was analyzed using the R package ggplot2 based on the TCGA and GTEx databases. **B** The correlation between drug sensitivity and the expression of SLC7A11, AIFM2, and GPX4 was analyzed using the Gene Set Cancer Analysis (GSCA) database. **C** The relationship between sorafenib and four ferroptosis-related genes in RCC samples. **D** The effect of expression of four ferroptosis-related genes on the half-maximal inhibitory concentration (IC50) value of sorafenib in RCC samples. The predicted chemotherapeutic response of each sample was analyzed using the R package pRRophetic according to the Genomics of Drug Sensitivity in Cancer (GDSC) database. The IC50 of the samples was assessed by ridge regression. The Wilcox test was used to compare and determine the significant difference between two different groups.

RCC is the most common malignant tumor of the renal parenchyma. RCC originates from the renal epithelium and accounts for more than 90% of kidney cancers. The expression of key regulators of ferroptosis, such as GPX4, SLC7A11, and FSP1, was significantly upregulated, but ACSL4 expression was obviously downregulated in three major types of RCC based on The Cancer Genome Atlas (TCGA) and the Genotype-Tissue Expression (GTEx) databases (Fig. 3A). Double knockdown of GPX4 and GPD2 synergistically inhibited tumor growth by exacerbating ferroptosis in vitro and in vivo [86]. The effects of erastin on 60 tumor cell lines in 8 tissues revealed that RCC cells were more susceptible to erastin-induced cell death than other cells [280]. Further studies indicated that erastin induced RCC cell death in association with typical parameters of ferroptosis, including ROS accumulation and decreased GPX4 expression, and these effects were reversed by antioxidants [54]. Moreover, the expression of SLC7A11 and FSP1 was significantly and positively correlated with some chemotherapy drugs, according to the CTPR database, including erastin and RSL-3 (Fig. 3B). Sorafenib was approved by the FDA for the second-line treatment of metastatic and advanced RCC. Sorafenib is not only a tyrosine kinase inhibitor but also an inducer of ferroptosis in some cancer cells; furthermore, its clinical efficacy in the treatment of RCC indirectly supports the involvement of ferroptosis in RCC. Notably, the results of bioinformatics analyses indicate that GPX4 and ACSL4 are positively and negatively correlated with the sensitivity of sorafenib in RCC, respectively (Fig. 3C). In addition, higher GPX4 expression is correlated with a higher IC50 value of sorafenib; however, higher ACSL4 expression corresponds to a lower IC50 value of sorafenib in RCC patients, indicating that these ferroptosis-related genes are closely related to drug sensitivity (Fig. 3D) [281–283]. In fact, the effect of sorafenib on ferroptosis induction is still controversial. In contrast to well-known system Xc- inhibitors such as erastin and sulfasalazine, sorafenib failed to induce ferroptosis in a series of cancer cells. Whether sorafenib induces ferroptosis in RCC cells and the identity of its target need to be further explored [284].

Clear cell renal cell carcinoma (ccRCC or KIRC) is the most common type of RCC. Silencing GPX4 reduced the synthesis of GSH and led to lipid peroxidation, which resulted in a significant decrease in the number of ccRCC cells [54]. Therefore, ferroptosis inducers, such as erastin, BSO, sulfasalazine, and sorafenib, directly or indirectly promote GSH depletion and induce ferroptosis to inhibit the development of renal cancer (Fig. 2) [2, 11, 285–288]. In hereditary leiomyomatosis and renal cell cancer (HLRCC), the inactivation of fumarate hydratase (FH) leads to the accumulation of much fumarate [289, 290]. As a result, a large amount of protein underwent acidification, GPX4 activity was decreased, and cells were more prone to ferroptosis. The density of renal cancer cells also affects the sensitivity of cells to ferroptosis, which is achieved by regulating the transcription regulator TAZ-mediated epithelial membrane protein 1 (EMP1)-NOX4 pathway [291]. Therefore, TAZ may be a potential therapeutic target for ferroptosis in patients with RCC. SLC7A11 also plays an important role in the development of RCC. Both p53 and BRCA1-associated protein 1 (BAP1) inhibit the expression of SLC7A11, thereby suppressing the development of RCC by promoting ferroptosis [15, 119, 292, 293]. MIT-domain containing protein 1 (MITD1) expression is upregulated in patients with ccRCC and is correlated with poor prognosis. Knockdown of MITD1 significantly decreased cell proliferation and migration and triggered ferroptosis through the TAZ/SLC7A11 pathway in ccRCC cells [294]. Chromophobe (Ch) renal cell carcinoma (ch-RCC or KICH) cells contain high levels of GSH and GSSG and exhibit higher sensitivity to ferroptotic inducers [295]. Gamma-glutamyl transferase 1 (GGT1), a membrane-bound extracellular enzyme, plays a key role in glutathione homeostasis. GGT1 was profoundly downregulated in ch-RCC cells, and the overexpression of GGT1 significantly inhibited cell growth, impeded the uptake of cystine and reduced cellular levels of GSH and GSSG [295].

With further research, an increasing number of ferroptosis inducers have been identified and developed to treat RCC. Artesunate (ART) promotes the degradation of ferritin and increases the concentration of iron by activating lysosomal function [296]. Salinomycin and its synthetic derivative AM5 promote the generation of ROS via lysosome-accumulated iron to induce ferroptosis [297]. In addition, arginine-capped manganese silicate nanobubbles (AMSNs) are nano-iron inducers that accelerate the depletion of GSH and cause the inactivation of GPX4, thereby inducing ferroptosis in cancer cells [298]. More importantly, according to our analysis, GPX4 and ACSL4 are significantly associated with various oncogenesis-related signaling pathways, such as the tumor proliferation signature pathway, the EMT, angiogenesis and the tumor inflammation signature pathway. In addition, the combination of ferroptosis inducers and commonly used chemotherapy drugs is a promising tumor treatment strategy [299–302]. Everolimus and erastin/RSL3 synergistically induce ferroptosis to reduce the viability of RCC cells through the inhibition of the mTOR-4EBP1 pathway; this result indicates that the combination of chemotherapy drugs with ferroptosis inducers may be used to overcome drug resistance [303]. Therefore, targeting ferroptosis may alleviate the drug resistance of renal cancer cells, reduce damage to normal cells, and contribute to guide individualized and precision drug therapy.

All the evidence indicates that ferroptosis is involved in the occurrence, progression and metastasis of renal cancer, and more therapeutic methods for renal cancer targeting ferroptosis can be continued into the future. However, the genetic determinants of ferroptosis in RCC cells remain unknown, and further studies are needed.

## Summary and outlook

In recent years, ferroptosis, as a novel form of cell death, has led to new ideas for the treatment of kidney diseases and drug development. Ferroptosis is a type of regulated cell death characterized by the accumulation of intracellular iron and lipid ROS. Since ferroptosis was first identified in 2012, an increasing number of studies have been focused on ferroptosis and kidney disease, but compared with that of other forms of death, the research on ferroptosis in kidney disease is still at an immature stage. It is urgent to further explore the mechanisms that regulate ferroptosis in different kidney diseases and determine how to effectively regulate ferroptosis. To date, most of the studies on ferroptosis in the field of kidney diseases have focused on AKI and RCC. The molecular mechanisms and precise roles of ferroptosis in chronic kidney diseases, such as renal fibrosis, diabetic kidney disease and polycystic kidney disease, need further investigation. Although iron metabolism abnormalities and lipid peroxidation mediated by ferroptosis are important mechanisms that regulate kidney diseases and ferroptosis-related defense mechanisms, including the system Xc-/GSH/GPX4 pathway, the mitochondrial pathway and the FSP1/CoQ_10_/NAD(P)H pathway, the specific details and crosstalk among these pathways are still not well understood. Regarding the mechanism of ferroptosis, the molecules that ultimately play a role in ferroptosis need to be further explored. The excessive accumulation of lipid peroxides, ROS and PUFAs in the plasma membrane leads to cell membrane damage or ferroptosis. Therefore, potential therapeutic targets for regulating ferroptosis by enzymes related to PUFA synthesis need to be studied further. Moreover, the mechanisms of ferroptosis regulation, such as iron transport and storage granules in iron corpuscles, and the correlation between ferroptosis and other human diseases are being further studied. Although many genes have been proven to cause ferroptosis, little is known about epigenetic modifications regulating the ferroptosis pathway. A recent study showed that the expression of lysine-specific demethylase 1 (LSD1) was increased in IR-treated AKI mice and H/R-treated HK-2 cells. Treatment with TCP, a specific inhibitor of LSD1, reduced the severity of IR-induced AKI by alleviating oxidative stress and ferroptosis. Moreover, pharmacological or genetic inhibition of LSD1 suppressed the TLR4/NOX4 signaling pathway to relieve AKI [304].

Exploring the molecular mechanisms underlying ferroptosis and identifying related signaling pathways in various kidney diseases will also elucidate new targets and directions for the research and development of kidney disease drugs. Significant progress has been made in inducing and inhibiting ferroptosis. Fer-1, a specific inhibitor of ferroptosis, has shown excellent protective and therapeutic effects in various animal kidney disease models. However, to do, there has been no clinical study of Fer-1. In the process of developing new inhibitors of ferroptosis, screening effective inhibitors from among a list of existing drugs is a quick and economical method. Thus, the regulatory effect of natural small molecule compounds on ferroptosis, especially on the treatment of kidney diseases, has attracted wide attention. As far as recent research results are concerned, the use of small-molecule drugs includes the treatment of kidney diseases, such as AKI, CKD, and RCC. For example, PA, ALR, promethazine, and natural small-molecule drugs, such as nuciferine, paricalcitol, QCT, and irisin, can be used to treat AKI because they upregulate GPX4 expression and activate GPX4-related pathways. In addition, natural small-molecule compounds can also alleviate AKI through different pathways, such as the lipid peroxidation and iron metabolism pathways. However, it should not be ignored that natural small-molecule compounds may also cause kidney damage by inducing ferroptosis. For example, patulin induces ferritinophagy-dependent cell death and then AKI [305]. In addition, some natural small-molecule compounds also exert dual regulatory effects on the ferroptosis pathway. For example, although nobiletin alleviates AKI in UUO mice by suppressing ferroptosis, it induces ferroptosis in melanoma cells through the GSK3β-regulated Keap1/NRF2/HO-1 pathway [248, 306]. These data reveal the complex mechanisms attributed to small-molecule compounds in regulating ferroptosis, and the results of contradictory regulatory mechanisms vary according to different cell types, specific environments and multiple human diseases. Therefore, in future preclinical and clinical trials, the nephrotoxicity of small-molecule compounds will be a concern that requires specific attention. Recently, cuproptosis, a novel RCD caused by excessive levels of cellular copper, was identified [307]. Cuproptosis and ferroptosis share some characteristics, such as increased density of the bilayer membrane structure, lethal ROS production due to the Fenton reaction and mitochondrion-regulated cell death. The questions of how cuproptosis regulates kidney diseases and whether ferroptosis and cuproptosis pathways intersect remain to be further explored. In summary, the in-depth study of ferroptosis will help promote the targeted prevention and treatment of kidney diseases in the clinic. These basic studies provide a solid theoretical basis for the treatment of kidney diseases and provide valuable information for the prevention and treatment of primary renal diseases and kidney complications.

## Data Availability

The expression datasets analyzed for this study can be downloaded from the TCGA database (https://portal.gdc.cancer.gov). The correlation between drug sensitivity and mRNA expression was analyzed using the Gene Set Cancer Analysis (GSCA) database (http://bioinfo.life.hust.edu.cn/GSCA/#/). The relationships between sorafenib and four ferroptosis-related genes in RCC were analyzed using the Genomics of Drug Sensitivity in Cancer (GDSC) database (https://www.cancerrxgene.org/). All data generated or analyzed during this study are included in this article. Further inquiries can be directed to the corresponding authors.
